# Retrospective Evaluation of Gastrointestinal Signs in Hypothyroid Dogs

**DOI:** 10.3390/ani13162668

**Published:** 2023-08-19

**Authors:** Eleonora Gori, Paola Gianella, Ilaria Lippi, Veronica Marchetti

**Affiliations:** 1 Veterinary Teaching Hospital “Mario Modenato”, Department of Veterinary Sciences, University of Pisa, Via Livornese Lato Monte, 56122 Pisa, PI, Italy; eleonora.gori@unipi.it (E.G.); veronica.marchetti@unipi.it (V.M.); 2 Department of Veterinary Medical Sciences, University of Turin, Largo Paolo Braccini 2, 10095 Grugliasco, TO, Italy; paola.gianella@unito.it

**Keywords:** canine, thyroid, intestinal, hepatic, gallbladder

## Abstract

**Simple Summary:**

Gastrointestinal signs are reported in humans with hypothyroidism, and resolution of GI signs was reported with thyroid hormone supplementation; however, GI involvement in the clinical presentation of dogs with hypothyroidism has not been investigated over the past decades. Our study aimed to fill this scientific gap, evaluating the prevalence and characteristics of concurrent gastrointestinal signs in hypothyroid dogs, describing laboratory and ultrasonographic findings and analyzing gastrointestinal signs after thyroid replacement therapy. Approximately 45% of hypothyroid dogs had gastrointestinal signs, especially constipation and diarrhea. At the abdominal ultrasound, gallbladder disease was present in more hypothyroid than euthyroid dogs. Finally, all hypothyroid dogs had a significant improvement in gastrointestinal signs after thyroid therapy. Our results, especially the improvement in clinical intestinal signs following thyroid therapy, support the association between gastrointestinal signs and hypothyroidism.

**Abstract:**

Few observations about gastrointestinal (GI) signs in hypothyroid dogs (hypo-T dogs) are available. We aimed to evaluate the prevalence and characteristics of concurrent GI signs in hypo-T dogs, describe clinicopathological, hepato-intestinal ultrasound findings in hypo-T dogs, investigate changes in GI signs after thyroid replacement therapy (THRT). Medical records of suspected hypo-T dogs from two hospitals were retrospectively reviewed. The inclusion criteria were: (1) having symptoms and clinicopathological abnormalities related to hypothyroidism (i.e., mild anemia, hyperlipemia); (2) not being affected by systemic acute disease; (3) not having received any treatment affecting thyroid axis. Hypothyroidism had to be confirmed using low fT4 or TT4 with high TSH and/or inadequate TSH-stimulation test response; otherwise, dogs were assigned to a euthyroid group. Clinical history, GI signs, hematobiochemical parameters, and abdominal ultrasound findings were recorded. Hypo-T dogs were assigned to the GI group (at least 2 GI signs) and not-GI group (1 or no GI signs). Follow-up information 3–5 weeks after THRT was recorded. In total, 110 medical records were screened: 31 dogs were hypo-T, and 79 were euthyroid. Hypo-T dogs showed a higher prevalence of GI signs (44%), especially constipation and diarrhea (*p* = 0.03 and *p* = 0.001), than euthyroid dogs (24%) (*p* = 0.04). Among hypo-T dogs, no difference in hematological parameters between GI and non-GI groups was found. Hypo-T dogs had a higher prevalence of gallbladder alterations than euthyroid dogs (20/25; 80% and 32/61; 52% *p* = 0.04). The hypo-T GI group showed a significant improvement in the GI signs after THRT (*p* < 0.0001). Specific investigation for concurrent GI diseases in hypo-T dogs was lacking; however, improvement in GI signs following THRT supports this association between GI signs and hypothyroidism.

## 1. Introduction

Hypothyroidism is one of the main endocrine disorders in dogs [1]. Lymphocytic thyroiditis and idiopathic thyroid atrophy are the most common causes of this condition, and clinical signs appear following the destruction of 75% of glandular parenchyma [1]. The most common clinical signs of hypothyroidism are related to decreased metabolic rate and dermatological changes, while less frequent but well-documented clinical manifestations include neurological, cardiovascular, and reproductive abnormalities [1]. In both human and veterinary medicine, hypothyroidism is a well-known cause of alteration of multiple gastrointestinal processes [1,2,3]. Patients can suffer mainly from constipation [3] as a result of abnormal peristalsis [4] due to alterations of hormonal receptors and neuromuscular disorders using mucopolysaccharides infiltration. However, chronic diarrhea has also been reported as a consequence of increased bacterial growth due to intestinal hypomotility [5,6,7]. Thyroid hormones are involved in several intestinal processes. In fact, the homeostasis of the intestinal epithelium is influenced by the interaction between T3 and intestinal thyroid receptors, specifically TR alpha 1 [8]. Human patients and experimental murine models with a lack of exhibition of intestinal TRα show gross morphologic abnormalities of intestinal epithelium and severe gastrointestinal symptoms [8]. Additionally, thyroid hormones are effectively metabolized to glucuronidated iodothyronines (T3G and T4G), which are rapidly eliminated through the intestine. Once in the intestine, T3G and T4G can be hydrolyzed back to T3 and T4 by the intestinal microbiota itself, suggesting that T3G and T4G serve as an intestinal thyroid hormone reservoir [8].

In human medicine, subclinical hypothyroidism may be associated with an increased risk of developing liver disease [3,9], especially Non-Alcoholic Fatty Liver Disease and fibrosis [10]. The increased risk of liver disease in hypothyroid patients has been associated with different mechanisms, such as dyslipidemia and higher body mass index, lack of intrahepatic lipolysis induced by thyroid hormones, decreased triglyceride clearance and increased hepatic accumulation of triglycerides, and lipogenesis induced by hypothyroidism-related insulin resistance [11]. In dogs, hypothyroidism has been reported as one of the differentials for glycogen-type vacuolar hepatopathy, defined as a reversible hepatocellular cytosolic vacuolation [12]. In addition, “hypothyroid hepatopathy” is described as a consequence of decreased circulating thyroid hormones; this can affect hepatic metabolism and cholesterol turnover in the liver [13]. On the other hand, gallbladder abnormalities are promoted by three different mechanisms: a decrease in bilirubin excretion rate due to lower activity of bilirubin UDP-glucuronyltransferase, impairing hepatic bilirubin metabolism, hypercholesterolemia, and hypotonia of the gallbladder causing delayed emptying of the biliary tract [14,15]. These mechanisms are also hypothesized in dogs, where an association with gallbladder abnormalities, specifically gallbladder mucocele, has been highlighted [16].

In humans, most of the gastrointestinal alterations often resolve with treatment of the underlying thyroid disease, thus confirming the hypothesis of a causal relationship [3,17,18,19]. In veterinary medicine, although often reported in textbooks [20], information regarding GI signs in hypo-T dogs is scarce [1,21,22,23,24,25,26]. An old and isolated experimental study demonstrated a decreased electrical and mechanical activity of the stomach and jejunum in four Labrador retrievers that underwent complete thyroidectomy [21]. In hypoT-dogs, an association with generalized, acquired megaesophagus has been reported. Polyneuropathy or myopathy associated with hypothyroidism may be responsible for megaesophagus, although the majority of reported cases did not show resolution of megaesophagus with thyroid replacement therapy [1,21,22,23,27]. In a recent case report, complete resolution of clinical and radiographic signs of megaesophagus was obtained with THRT [23]. Based on these premises, this study aimed: (1) to evaluate the prevalence and characteristics of concurrent GI signs in dogs with hypothyroidism, compared to a population of euthyroid dogs; (2) to describe clinicopathological, intestinal and hepatic ultrasound findings in these two groups; and (3) to analyze changes in the frequency of GI signs after THRT in a population of hypo-T dogs.

## 2. Materials and Methods

In this retrospective study, the electronic medical record database of all canine patients admitted at two different Veterinary Teaching Hospitals (University of Pisa, 2009–2022; and University of Turin, 2011–2022) was searched to identify dogs tested for hypothyroidism based on compatible clinical signs (e.g., increased body weight, weakness, lethargy, exercise intolerance) and clinicopathological abnormalities (e.g., anemia, hyperlipemia, increased liver enzymes). Only medical records of dogs that had an evaluation of thyroid function were considered. The inclusion criteria for the classification of a dog as having hypothyroidism (hypo-T) were the following: (1) having symptoms and clinicopathological abnormalities related to the hypothyroidism that justify specific diagnostic tests; (2) having the disease confirmed through low fT4 or TT4 values with high TSH values and/or inadequate TSH-stimulation test response [24]; (3) not being affected by any systemic acute disease [24]; (4) not having received any treatment affecting thyroid axis (e.g., glucocorticoids, phenobarbital, amiodarone, phenylbutazone, sulfonamides, …) [24]. Dogs with results of fecal flotation, complete hematobiochemical profile, serum folate, and cobalamin concentrations, pancreatic specific lipase levels, serum basal cortisol, and C-reactive protein concentrations suggestive of extra-thyroidal diseases were ruled out. An inadequate TSH-stimulation test response was confirmed by a pre- and post-TT_4_ concentration below the reference range (<1.5 μg/dL) or a post-TT_4_ less than 1.5-fold the basal TT_4_ concentration [24]. A TSH-stimulation test was performed in any other case than concurrent high TSH values and low ft4 or TT4 values (i.e., normal TSH + low fT4). Parameters of the thyroid profile were assessed in different laboratories but using the same chemiluminescence methodology. Dogs that fulfilled the above inclusion criteria (compatible clinical and hematological signs and specific thyroid function tests confirming hypo-T) were included in the hypo-T dogs’ group. Dogs with clinical signs suggestive of hypo-T but normal or incompatible specific thyroid function tests were assigned to the euthyroid group.

Information concerning the presence of GI signs, their characterization, and frequency before and after 3 to 5 weeks of THRT was also recorded. Briefly, the following aspects of GI signs were considered during the medical records review: (1) presence of vomiting (frequency, duration, relationship with meal or water intake, appearance, presence or absence of blood or bile) and/or nausea (refuse to eat, hypersalivation, chewing and search and ingestion of grass) [28]. Based on its duration, vomiting was also differentiated into acute (few days onset) or chronic (persistent for more than 1–2 weeks) [29]; (2) presence of diarrhea [defined as an increased frequency (>3/die), and/or decreased consistency of feces (fecal score 6–7/7; Purina Fecal scoring system; [30], and/or increased volume of feces [31]. Diarrhea was also distinguished, based on its duration in acute (few days onset) or chronic, if persistent for more than 3 weeks or intermittently present for more than 3 weeks [31]. Based on diarrhea-associated clinical signs and appearance of feces, diarrhea was classified as small bowel diarrhea (frequency of defecation: normal to mildly increased; fecal volume: normal to increased; fecal mucus: usually absent; tenesmus, urgency: absent; weight loss: common) and large bowel diarrhea (frequency of defecation: markedly increased; fecal volume: decreased; fecal mucus: often present; tenesmus, urgency: often present; weight loss: uncommon) [31]. If this distinction was not possible and dogs had signs of both small and large bowel diarrhea, they were identified as “mixed diarrhea”; (3) the presence of constipation was defined as infrequent or difficult defecation of dry and hard stools [32]. Constipation criteria were frequency of defecation less than once a day, increased consistency of stool (score of 1–2/7) (Purina Fecal Scoring System [32]); (4) presence of flatulence; (5) episodes of abdominal pain connected to the GI sign.

Frequency of the above GI signs was graded from 0 to 5 points, based on Poncet et al. study [33]: 0 (never/absent), 1-occasionally (less than 1 episode per month), 2-regularly (once a week), 3-daily (once a day), 4-often (more than once a day) and 5-constantly.

Based on the above-reported GI signs, dogs were then divided into two groups: the non-GI group (dogs without GI signs or with only one GI sign) and the GI-group (dogs with at least 2 GI signs). Subsequently, hematobiochemical analyses performed at the time of the diagnosis of hypo-T were reviewed, and the presence of alterations commonly associated with hypo-T was recorded: anemia, hypercholesterolemia, and hypertriglyceridemia, increased liver enzymes (alanine aminotransferase, ALT; alkaline phosphatase, ALP; gamma-glutamyltransferase, GGT) and total bilirubin (TBIL). In addition, serum levels of total proteins (TP) and albumin (ALB) were recorded.

If available, abdominal ultrasound findings at the time of hypo-T diagnosis were also reviewed, with particular attention to gastrointestinal and/or hepatic involvement. Abdominal ultrasound was considered suggestive of intestinal disorder in case of abnormal intestinal stratigraphy and thickness and/or alterations of mucosal echogenicity, associated or not with mesenteric lymph node enlargement [34]. Ultrasound signs of hepatomegaly, abnormal liver echogenicity, and/or gallbladder alteration, and the presence of echogenic content within the gallbladder and dilation of the bile duct were considered suggestive of liver disorder [35]. Data regarding general metabolic and GI signs collected at clinical follow-up 3 to 5 weeks from the beginning of THRT were also reviewed. Dogs receiving additional therapies or specific diets after THRT were excluded.

### Statistical Analysis

Statistical analysis was performed using commercial statistical software (IBM SPSS Statistics, version 25, IBM Corporation, New York, NY, USA). As reported above, the study population was divided into GI and non-GI hypo-T dogs to evaluate the prevalence of GI signs in the hypo-T dog population. Secondly, for the evaluation of hematobiochemical alterations (presence/absence of anemia, increased liver enzymes, hypercholesterolemia, hypoalbuminemia, hypoproteinemia, hypertriglyceridemia, and hyperbilirubinemia) and abdominal ultrasound abnormalities (presence/absence of degenerative liver disease, gallbladder sludge/mucocele, small and large bowel involvement) were compared between GI and non-GI dogs, using Pearson Chi-square test or Fisher’s exact test. Finally, to observe the trend of gastrointestinal signs in the GI group, pre- and post-THRT prevalence of GI signs was compared using the McNemar test. A *p*-value < 0.5 was considered statistically significant.

## 3. Results

### 3.1. Study Population

A total of 110 medical records of dogs with suspected hypothyroidism were initially screened: 31 dogs filled the inclusion criteria and were considered hypo-T, 79 were considered euthyroid, and were used as a control group. Among hypo-T dogs, 21 showed low T4 or fT4 and high TSH values. TSH stimulation test was performed in 10 dogs, 2 of which underwent the TT4 and TSH evaluation. Of these latter two dogs, tT4 was 0.5 μg/dL in both dogs, and TSH was 0.03 and 0.15 μg/dL, respectively. Among hypo-T dogs, 23 (74%) were females (20 spayed), and 8 (26%) were males (2 neutered). Median age was 9 years (range 1–15 years). Six dogs were mixed breed, and the remaining 25 dogs belonged to various breeds: Golden Retriever and Cocker Spaniel (3 dogs each breed), Dachshund and Miniature Schnauzer (2 dogs each breed), Akita Inu, Beagle, Border Collie, Epagneul Breton, Cavalier King Charles Spaniel, Dobermann Pinscher, Galgo Espanyol, Lagotto Romagnolo, Maltese, Shepherd Maremma, Pinscher, English Setter, Italian Spinone, and English Springer Spaniel (1 dog each breed).

The control group of euthyroid dogs was composed of 79 dogs, 62 of which (79%) were females (53 spayed), and 17 were males (5 neutered). Median age was 9.9 years (range 0.4–20 years). Twenty-eight dogs were mix-breed, whereas the remaining 51 dogs were purebreds: Cavalier King Charles spaniel and German Shepherd (5 dogs each), Labrador Retriever, Miniature Schnauzer, Golden Retriever (4 dogs each), Poodle and Jack Russel Terrier (3 dogs each), West Highland White Terrier, Yorkshire Terrier and English Bulldog (2 dogs each), American Staffordshire Terrier, Beagle, Border Collie, Boxer, Bull Terrier, Dogo Argentino, Galgo Espanyol, Greater Swiss Mountain Dog, Lagotto Romagnolo, Maltese, Shepherd Maremma, Australian Shepherd, Rhodesian Ridgeback, Italian Hound, English Setter, Shar Pei, Spitz (1 dog each breed). Median (min-max range) values of fT4, tT4, and TSH were 10.5 nmol/L (0.9–36), 1.9 μg/dL (0.5–33.6), and 0.2 nmol/L (0.04–1.2), respectively.

### 3.2. Prevalence and Characteristics of Concurrent GI Signs in Hypo-T Dogs Compared to Euthyroid Dogs

Hypo-T dogs showed a higher prevalence of GI signs (44%) compared to euthyroid dogs (24%) (*p* = 0.04). The prevalence of vomiting, nausea, flatulence, and abdominal pain was not significantly different between euthyroid and hypo-T dogs (all *p*-values > 0.05). Hypo-T dogs showed a higher prevalence of diarrhea (*p* = 0.001) and constipation (*p* = 0.03) (47% and 13%, respectively) compared to euthyroid dogs (17% and 3%, respectively). In hypo-T dogs, diarrhea was chronic in 12 dogs (80%) and acute in 3 dogs (20%). Ten dogs had large bowel diarrhea, while five dogs had mixed diarrhea. Vomiting was chronic in all five dogs that showed it. Of the 31 hypo-T dogs, 14 dogs showed at least 2 GI signs among the above-mentioned ones and were included in the hypo-T GI group. The remaining 17 dogs were classified as hypo-T non-GI groups. GI signs and their characteristics in the study population are reported in Table 1.

### 3.3. Clinicopathological Intestinal and Liver Parameters of Hypo-T Dogs with and without Gastrointestinal Signs

Median and ranges of liver enzymes (ALT, ALP, and GGT) and total bilirubin (TBIL), as well as serum total proteins (TP) and albumin (ALB) and their prevalence are reported in Table 2. No differences in GI and non-GI dogs between the selected variables were found (all *p*-values > 0.05).

### 3.4. Ultrasonographic Intestinal and Liver Features of Hypot-Dogs with and without Gastrointestinal Signs and Comparison with Euthyroid Group

Abdominal ultrasound was available for review in 25 out of 31 hypo-T dogs. Intestinal and liver ultrasonographic abnormalities in our hypothyroid cohort are reported in Table 3. Of the 79 euthyroid dogs, 61 had an abdominal ultrasound available for review. Intestinal involvement was present in 19 euthyroid dogs (31%), and there was no significant difference with the hypo-T dogs (19%; *p* = 0.3). Thirty-nine euthyroid dogs (65%) had an ultrasound liver abnormality, although the prevalence of the liver abnormality was not significantly different compared to hypo-T dogs (66%; *p* = 0.9). Finally, hypo-T dogs showed a higher prevalence of gallbladder abnormalities compared to euthyroid dogs (20/25; 80% and 32/61; 52% *p* = 0.04). Based on the clinical and ultrasonographic findings, 19 dogs had intestinal involvement, 11 of which of the large bowel, and the remaining 8 of the small bowel. No dogs showed dilation of the bile duct. No differences in the prevalence of ultrasonographic abnormality in the GI and non-GI group was found (all *p*-values > 0.05).

### 3.5. Prevalence of Gastrointestinal Signs Follow-Up after THRT in Hypo-T Dogs

Each of the 31 hypo-T dogs had a re-check after the start of the THRT. Twenty-three of 31 dogs had only the THRT, whereas the other three dogs had both a dietary change to a gastrointestinal commercial diet and the THRT. There was a significant improvement in the GI signs in hypo-T GI dogs, where all dogs had a significant improvement in clinical GI signs (*p* < 0.0001). Resolution of GI was observed in all hypo-T dogs.

## 4. Discussion

Gastrointestinal signs in hypo-T dogs have been anecdotally reported, although no specific clinical studies have been conducted. Therefore, this study aimed to describe the concurrent intestinal and liver involvement in dogs with hypothyroidism.

Compared to euthyroid dogs, a higher prevalence of GI signs, especially chronic, was found in hypo-T dogs. Diarrhea, nausea, and vomiting were the most represented clinical signs, followed by constipation and dyschezia, both of which were significantly associated with the presence of hypothyroidism. In our hypo-T dogs, clinical signs, such as diarrhea, constipation, and/or dyschezia, and the presence of mucoid feces were indicative of large bowel involvement. This finding is also supported by the fact that large bowel symptoms are among those commonly present in human hypothyroid patients [36]. In particular, the significant presence of diarrhea and constipation in hypo-T dogs may support the hypothesis of an association between gastrointestinal signs and abnormal peristalsis, mediated using thyroxine [36], as already reported in humans. In hypothyroid humans, dysmotility of the gastrointestinal tract is known to cause dysphagia, esophagitis, esophageal reflux, hiatal hernia, dyspepsia, constipation, and, in the most serious cases, pseudo-obstruction of the colon, megacolon, paralytic ileus, and intestinal ischemia [3,36]. Abnormal peristalsis may also be due to a secondary intestinal dysbiosis [5,37], as well as the presence of diarrhea and flatulence may be expression of it. In fact, the onset of diarrhea following small intestinal bacterial overgrowth in six hypothyroid dogs has already been reported in a previous study by Jackson and colleagues [38]. Indeed, the presence of intestinal dysbiosis, defined as a qualitative/quantitative alteration of the intestinal microbiota, may be secondary to alterations of the intestinal function or its motility [37], as already reported in hypothyroidism [39,40,41]. Specifically, as intestinal microbiota has been demonstrated essential in the hydrolysis of the glucuronidated forms of T3 and T4, any modification of it may cause an alteration in the thyroid hormones’ homeostasis and reservoir [7].

Although ultrasonographic signs of liver disease, gallbladder, and intestinal involvement were found in hypo-T dogs, no statistically significant difference was reported compared to euthyroid dogs. This high prevalence of signs of liver disease may suggest a pathogenetic contribution of the liver to the GI signs, with dysbiosis as a possible connecting element. Although dysbiosis may be primarily caused by dysmotility [5,37], it is also possible that it is secondary to qualitative/quantitative alterations of the bile [42], which was a frequent finding in our hypo-T dogs (approximately 60%). On the other hand, dysbiosis may contribute to reactive hepatopathy by producing microbial-derived toxins and other metabolites [43].

The presence of gallbladder disease, defined as alterations of the gallbladder wall, presence of echogenic content inside it, and/or dilatation of the bile duct, was a frequent finding in our hypo-T dogs (approximately 63% of dogs), which has already been associated with thyroid hormone deficiency in the literature. Hepatobiliary alterations may also be caused by dyslipidemia, impaired hepatic metabolism, altered bile composition, and increased tonicity of the sphincter of Oddi [3,44,45]. Therefore, this study supports a possible association between hypothyroidism and alterations of the liver and gallbladder, which deserves to be taken into consideration by the clinician, especially due to the potential implications in determining the onset of gastrointestinal signs.

Furthermore, an improvement in the GI signs following the administration of levothyroxine was observed at follow-up in hypo-T dogs. In our opinion, these findings may suggest a possible relationship between hypothyroidism and gastrointestinal signs, as previously supported by numerous human studies. These studies support the role of the lack of thyroid hormones in causing gastrointestinal alterations [5,37,42]. This relationship has also been reported in human literature by showing a resolution of GI clinical signs following treatment for hypothyroidism [5,36,44,45,46].

This study has several limitations. The first limitation is due to the small number of recruited hypo-T dogs, which might underpower the statistical analysis of some data. A second limitation is linked to the possible classification of some hypo-T dogs as euthyroid dogs, since not all euthyroid dogs had a TSH stimulation test, which is considered the gold standard for diagnosis of canine hypothyroidism. A final limitation is the lack of specific investigations for concurrent GI and pancreatic diseases, which might lead to hypothyroid dogs with a primary gastrointestinal and pancreatic disease. This might depend on several factors, such as the owner’s difficulty in defining the onset of clinical signs or in recognizing mild gastrointestinal signs, especially for outdoor dogs.

## 5. Conclusions

Our study identified a high prevalence of gastrointestinal signs, especially constipation and diarrhea, in hypothyroid dogs. The improvement in gastrointestinal signs following treatment with levothyroxine supports a possible association between gastrointestinal signs and hypothyroidism, although the pathogenetic mechanisms need to be further clarified. In addition, intestinal dysbiosis may also be the consequence of loss of balance in the liver-gut axis, given that many dogs here had biochemical and ultrasonographic signs of hepatobiliary involvement. However, these results should be interpreted with caution since the concurrent presence of hypothyroidism and primary gastrointestinal or pancreatic diseases could not be ruled out. Future studies are needed to explore this interesting topic.

## Figures and Tables

**Table 1:** Gastrointestinal clinical signs and their characteristics in the study population.

Clinical Signs	Hypo-T Dogs (*n* = 31)		Euthyroid Dogs (*n* = 79)		*p*-Value
	Prevalence (%)	Frequency ^1^	Prevalence (%)	Frequency ^1^	
Diarrhea *	15 (47%)	1 → 4 dogs 2 → 4 dogs 3 → 3 dogs 4 → 1 dog 5 → 3 dogs	14 (18%)	1 → 6 dogs 2 → 3 dogs 3 → 4 dogs 5 → 1 dog	0.001
Nausea	10 (31%)	1 → 8 dogs 2 → 1 dog 3 → 1 dog	26 (33%)	1 → 14 dogs 2 → 6 dogs 3 → 4 dogs	0.8
Flatulence	9 (28%)	2 → 4 dogs 3 → 4 dogs 4 → 1 dog	12 (15%)	1 → 4 dogs 2 → 5 dogs 4 → 3 dogs	0.12
Vomiting	5 (17%)	1 → 4 dogs 2 → 1 dog	17 (22%)	1 → 9 dogs 2 → 7 dogs	0.6
Constipation *	5 (17%)	1 → 1 dog 2 → 2 dogs 3 → 1 dog 5 → 1 dog	3 (3%)	1 → 1 dog 2 → 1 dog	0.03
Abdominal pain	3 (9%)	1 → 2 dogs 3 → 1 dog	4 (5%)	1 → 3 dogs 2 → 1 dog	0.4

^1^ Frequency based on Poncet et al. [33] study: 0 (never/absent), 1 (less than 1 episode per month), 2 (once a week), 3 (once daily), 4 (more than once daily) and 5 (constantly). *, statistically significant.

**Table 2:** Median and ranges of liver enzymes, total protein, and albumin in 31 hypothyroid dogs.

Parameters	Non-GI Group (*n* = 17)		GI Group (*n* = 14)		Reference Interval
	Median (range)	Prevalence of ↑/↓	Median (range)	Prevalence of ↑/↓	
ALT (U/L)	67 (9.5–226)	↑ 9 (50%)	55 (20–115)	↑ 3 (22%)	20–70
ALP (U/L)	144 (22–1175)	↑ 4 (22%)	108 (43–3640)	↑ 5 (35%)	45–250
GGT (U/L)	5.3 (1.8–46.7)	↑ 5 (27%)	4 (1–17.2)	↑ 1 (7%)	2–11
TBIL (mg/dL)	0.22 (0.08–0.48)	↑ 2 (11%)	0.16 (0.1–0.28)	↑ 0 (0%)	0–0.3
COL (mg/dL)	448 (214–1146)	↑ 10 (55%)	340 (166–888)	↑ 10 (72%)	120–280
TRI (mg/dL)	174 (58–750)	↑ 10 (55%)	349 (59–757)	↑ 9 (64%)	25–90
TP (g/dL)	7 (5.6–8.3)	↓ 1 (3%)	6.7 (4.8–8)	↓ 1 (7%)	5.8–7.8
ALB (g/dL)	3.2 (2.2–4)	↓ 1 (3%)	3.4 (2.6–4.4)	↓ 3 (22%)	2.6–4.1

ALT, alanine aminotransferase; ALP, alkaline phosphatase; COL, cholesterol; GGT, gamma-glutamyltransferase; TBIL, total bilirubin; TP, total protein; TRI, triglycerides; ALB, albumin, RI, reference interval; ↓ parameter lower than RI; ↑ parameter above the RI.

**Table 3:** Intestinal and liver ultrasonographic abnormalities in 31 hypothyroid dogs.

Ultrasonographic Abnormality	Prevalence	Characteristics
Intestinal stratigraphy/ thickness	4/25 (15%)	Colon → 3 dogs Jejunum → 1 dog
Intestinal mucosa echogenicity	1/25 (3%)	Colon → 1 dog
Enlarged abdominal lymph nodes	3/25 (11%)	
Hepatomegaly	11/25 (42%)	
Liver parenchyma echogenicity	17/25 (65%)	
Gallbladder wall thickness	9/25 (34%)	
Gallbladder echogenic content	20/25 (77%)	

## Data Availability

Data will be available upon reasonable request.
